# The health impact of the Triple Challenge of Brexit, COVID-19 and Climate Change in Wales: Liz Green

**DOI:** 10.1093/eurpub/ckac131.542

**Published:** 2022-10-25

**Authors:** L Green

**Affiliations:** Wales HIA Support Unit, Public Health Wales, Wrexham, UK

## Abstract

The COVID-19 pandemic has revealed the complex relationships between health, well-being, economy and society. The United Kingdom’s (UK) withdrawal from the European Union (Brexit) and climate change are having a cumulative, magnifying impact. UK nations have to tackle the multifaceted nature of Brexit, COVID-19 and climate change (‘Triple Challenge’ or TC) not only in isolation, but as a cumulative whole.This study provides a strategic overview of the interactions of the TC on determinants of health and equity in Wales and proposes opportunities to take forward. Using findings from existing Welsh Health Impact Assessments (HIAs) undertaken on the single challenges, rapid searches of the academic and grey literature were undertaken to identify evidence which focused on the TC and its impacts.From this, key determinants impacted and spotlight examples were identified and analysed. Evidence indicates the TC will have a wide range of compounding impacts across multiple determinants and inequalities. These will need to be viewed in synergy, not singularly. Determinants affected include i.e mental well-being, food insecurity, employment.Population groups potentially affected include rural communities, fishers/farmers, young people. Policy responses need to be constructed in an integrated way with cross sector involvement as actions intended to have positive impacts for one challenge could also have negative unintended impacts for others. This innovative work has highlighted the significant interconnectedness of the challenges. Developing an overarching policy approach could support lasting change.Having the TC as the underpinning focus point for new policies and strategies will help to maximise impact when addressing concerns in relation to post Brexit policy/actions, COVID recovery and climate change adaptation/mitigation.This work can be utilized by other nations as an example for challenges they may face in their context and nations in the UK/Europe affected by the TC.

**Key messages:**

• Brexit, COVID-19 and climate change have cumulative health impacts which must be considered together.

• Health impact assessment can provide a core framework through which to explore inequalities and health impact of multiple policy and practical issues.

